# On the interference alignment with limited feedback for device-to-device networks

**DOI:** 10.1186/s13638-016-0574-8

**Published:** 2016-03-03

**Authors:** Yang Zhang, Chenglin Zhao, Jusheng Yu, Li Hong, Hongxia Zhang

**Affiliations:** School of Information and communication Engineering, Beijing University of Post and Telecommunications (BUPT), Xï Tu Cheng Road 10, Beijing, 100876 China; School of Electronic Engineering, Beijing University of Post and Telecommunications (BUPT), Xï Tu Cheng Road 10, Beijing, 100876 China; Department of Disaster Prevention Instrument, Institute of Disaster Prevention, Xüeyuan Street, Sanhe, 101601 China; College of Computer and Communication Engineering, China University of Petroleum (UPC), Cḧangjiang xi Road 66, Qingdao, 266555 China

**Keywords:** Device-to-device communication, Interference alignment, Limited feedback, MIMO

## Abstract

In this paper, we analyze the performance of interference alignment of device-to-device (D2D) uplink underlay cellular networks. By fully considering the impact of imperfect precoding vectors caused by limited feedback, the exact closed-form expressions of average throughput for cellular network and D2D network are derived in terms of transmit power and the number of feedback bits. The accuracy of the average throughput is verified by simulation results. Our analytic results provide great promises to practical system designs.

## Introduction

Interferences in radar sensor networks and wireless communications have been one of the challenging problems. In radar sensor networks, the interference around the target degrades the detection capabilities of radars significantly, and various methods are proposed to suppress the sidelobe interference [1, 2] or mainlobe interference [3, 4]. For wireless sensor networks, interference reduces the throughput of networks and, therefore, restricts the spectral efficiency seriously [5, 6].

As a promising precoding technology, interference alignment is able to achieve the maximum degrees of freedom (DoF) by aligning all the interference into a lower-dimensional signal subspace at each receiver, which, therefore, has been paid great attention by academe [7]. In radar sensor networks, interference alignment in radar sensor networks such as orthogonal frequency offset [8–10] and opportunistic sensing in space [11, 12] have been very successful. In wireless communications networks, interference alignments considering parameters in frequency, time, and space could be studied as well [13–16].

Device-to-device (D2D) communication is introduced to increase the throughput of local wireless data transmission by enabling direct communications between physically proximal devices [17], which also coincide with some scenarios of wireless sensor networks. However, the interference caused by D2D devices reduces the performance of cellular communication significantly. Hence, how to eliminate the interference to cellular communications has become one of the most challenging works for D2D communication networks. In this paper, we analyze the performance of interference alignment of D2D uplink underlay cellular networks.

To reduce or eliminate the interference of D2D networks, Lu Yang et al. propose two interference alignment schemes to manage the interference between D2D communications and cellular communications [18]. The first one is proposed to align the interference signals from D2D users (DUs) into the orthogonal space of cellular communications at the base station and, alternatively, the other one aims to control the peak interference. Among the two interference alignment schemes, globally perfect channel state information (CSI) is assumed to be available in the base station, and precoding vectors are fed back to users without error. In practical scenarios, however, acquiring perfect CSI and/or feeding back perfect precoding vectors is infeasible due to the low-rate feedback links. So, the issues of limited feedback on multiple-input, multiple-output (MIMO) networks are widely studied in the research community [19, 20]. To the best of our knowledge, however, there is no reported work on interference alignment with limited feedback in the emerging D2D underlay networks.

In this work, we consider a practical system model consisting of a D2D local area networks (LAN) and a cellular uplink [18], where multiple DUs intend to communicate with a D2D receiver (DR). Under the assumption that the quantized precoding vectors are fed back to users through low-rate feedback links, we analyze the average throughput as a function of the number of feedback bits and transmit power and derive the closed-form expressions of throughput for cellular network and D2D network. Finally, we validate our analytic results by simulations.

*Notations*: Matrices and vectors are represented by bold uppercase and lowercase letters, respectively. Inversion, conjugation, transpose, and hermitian transpose are denoted by (·)^−1^, (·)^∗^, (·)^T^, (·)^*†*^, respectively. **A**⊗**B** is the Kronecker product of matrices **A** and **B**. The acronym i.i.d. means “independent and identically distributed.”

## System model

**Fig. 1:**
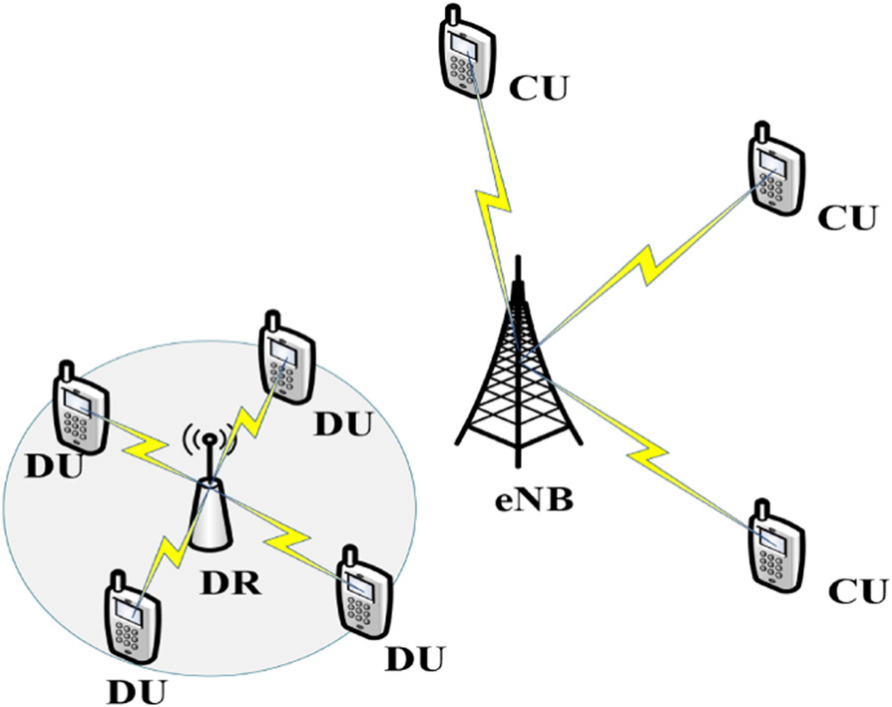
System model

As shown in Fig. 1, we consider a single-cell MIMO uplink network with a base station (BS). The BS is assumed to be equipped with *M*_*c*_ receiving antennas and supporting data traffic from *s* cellular users (CUs) simultaneously. Meanwhile, *l* other users are selected to communicate with a DR which is equipped with *M*_*d*_ receive antennas as DUs. Each user is equipped with *N* transmit antennas. For simplicity, we consider the system where each user transmits only a single data stream.

Assume that $\mathcal {Q}_{\text {CU}}$ and $\mathcal {Q}_{\text {DU}}$ represent the sets of CUs and DUs, respectively. Denote the large-scale fading gain from user $i \in \mathcal {Q}_{\text {CU}} \bigcup \mathcal {Q}_{\text {DU}}$ to BS and DR as ${\eta _{i}^{c}}$ and ${\eta _{i}^{d}}$ and the small-scale fading channel matrix from user *i* to BS and DR as $\mathbf {H}_{i}^{c}$ and $\mathbf {H}_{i}^{d}$, respectively. All fading coefficients are assumed to be i.i.d. circularly symmetric complex Gaussian random variables with zero mean and unit variance, i.e., $\mathcal {CN}(0,1)$. The signal at BS can be written as follows:

(1) $$ \begin{aligned} \mathbf{y}^{c} =& \sum_{i \in \mathcal{Q}_{\text{CU}}} \sqrt{P_{c} {\eta^{c}_{i}}} \left(\mathbf{U}^{c}\right)^{\dag} \mathbf{H}^{c}_{i} \mathbf{v}_{i} x_{i}\\ &+ \sum_{k \in \mathcal{Q}_{\text{DU}}} \sqrt{P_{d} {\eta^{c}_{k}}} \left(\mathbf{U}^{c}\right)^{\dag} \mathbf{H}^{c}_{k} \mathbf{v}_{k} x_{k} + \left(\mathbf{U}^{c}\right)^{\dag}\mathbf{z}_{c}, \end{aligned}  $$

and the signal at DR is given by the following:

(2) $$ \begin{aligned} \mathbf{y}^{d} =& \sum_{i \in \mathcal{Q}_{\text{CU}}} \sqrt{P_{c} {\eta^{d}_{i}}} \left(\mathbf{U}^{d}\right)^{\dag} \mathbf{H}^{d}_{i} \mathbf{v}_{i} x_{i} \\&+ \sum_{i \in \mathcal{Q}_{\text{DU}}} \sqrt{P_{d} {\eta^{d}_{k}}} \left(\mathbf{U}^{d}\right)^{\dag} \mathbf{H}^{d}_{k} \mathbf{v}_{k} x_{k} + \left(\mathbf{U}^{d}\right)^{\dag}\mathbf{z}_{d}, \end{aligned}  $$

where $\mathbf {U}^{c} = \left [\mathbf {u}^{c}_{1},\mathbf {u}^{c}_{2},\cdots,\mathbf {u}^{c}_{s}\right ] \in \mathbf {C}^{M_{c} \times s}$ and $\mathbf {U}^{d} = \left [\mathbf {u}^{d}_{1},\mathbf {u}^{d}_{2},\cdots,\mathbf {u}^{d}_{l}\right ] \in \mathbf {C}^{M_{d} \times l}$ denote the post-processing matrices of BS and DR, with $\left \|\mathbf {u}_{i}^{c}\right \|=\left \|\mathbf {u}_{j}^{d}\right \|=1$. **v**_*i*_∈**C**^*N*×*l*^ denotes the precoding vector of user *i*. *P*_*c*_ and *P*_*d*_ denote the transmit power of CUs and DUs, respectively. *x*_*i*_ denotes the data symbol of user *i* with **E**[∥*x*_*i*_∥]=1. **z**_*c*_ and **z**_*d*_ are the additive white Gaussian noise (AWGN) at BS and DR, respectively.

For convenience, it is assumed that the channels from all users are perfectly estimated at BS and DR. With perfect CSI, BS designs the post-precoding matrix **U**^*c*^ and precoding vectors for all CUs and DUs using the “interference-free” interference alignment scheme proposed in [18]. To feed back the precoding vectors from BS to users, the precoding vector **v**_*i*_ is quantized to $\bar {\mathbf {v}}_{i}$ with *B*_*i*_ bits random codebook, $\forall i \in \mathcal {Q}_{\text {CU}} \bigcup \mathcal {Q}_{\text {DU}}$. Following the theory of random vector quantization [19], the relationship between the original precoding vector **v**_*i*_ and the quantized precoding vector $\bar {\mathbf {v}}_{i}$ can be expressed as follows:

(3) $$ \bar{\mathbf{v}}_{i} = \cos(\theta_{i})\mathbf{v}_{i} + \sin(\theta_{i})\hat{\mathbf{v}}_{i},  $$

where $\theta _{i} = \angle (\mathbf {v}_{i}, \bar {\mathbf {v}}_{i})$ and sin(*θ*_*i*_) is the magnitude of the quantization error. $\hat {\mathbf {v}}_{i}$ is an unit norm vector isotropically distributed in the null space of **v**_*i*_.

Due to the quantization error, the interference that come from CUs cannot be eliminated completely with quantized precoding vectors. Therefore, the resulting residual interference power will degrade the throughput significantly. The achievable throughput of BS with limited feedback can be written as follows:

(4) $$ R_{\Sigma}^{c} = \sum_{i \in \mathcal{Q}_{\text{CU}}} \log_{2} \left(1 + \frac{P_{c}{\eta^{c}_{i}} \left| \left(\mathbf{u}_{i}^{c}\right)^{\dag} \mathbf{H}_{i}^{c} \bar{\mathbf{v}}_{i} \right|^{2}}{I_{i}^{c,c} + I_{i}^{c,d} + 1}\right),  $$

where $I_{i}^{c,c} = \sum _{n \in \mathcal {Q}_{\text {CU}}, n \neq i} P_{c} {\eta _{n}^{c}} \left | \left (\mathbf {u}_{i}^{c} \right)^{\dag } \mathbf {H}_{n}^{c} \bar {\mathbf {v}}_{n} \right |^{2}$ and $I_{i}^{c,d} = \sum _{k \in \mathcal {Q}_{\text {DU}}} P_{d} {\eta _{k}^{c}} \left | \left (\mathbf {u}_{i}^{c} \right)^{\dag } \mathbf {H}_{k}^{c} \bar {\mathbf {v}}_{k} \right |^{2}$ are the residual interference power from other CUs and DUs to the user *i*, respectively.

Finally, the DR designs its post processing matrix **U**^*d*^ with the quantized precoding vectors to eliminate the interference from other DUs perfectly, while treating the interference from cellular links as noise [18], yielding the following:

(5) $$ \left(\mathbf{u}_{k}^{d} \right)^{\dag} \mathbf{H}_{i}^{d} \bar{\mathbf{v}}_{i} = 0, \forall i,k \in \mathcal{Q}_{\text{DU}}, i \neq k.  $$

Hence, the throughput of D2D network is given by the following:

(6) $$ R_{\Sigma}^{d} = \sum_{k \in \mathcal{Q}_{\text{DU}}} \log_{2} \left(1 + \frac{P_{d}{\eta^{d}_{k}} \left| \left(\mathbf{u}_{k}^{d}\right)^{\dag} \mathbf{H}_{k}^{d} \bar{\mathbf{v}}_{k} \right|^{2}}{I_{k}^{d,c} + 1}\right),  $$

where $I_{k}^{d,c} = \sum _{i \in \mathcal {Q}_{\text {CU}}} P_{c} {\eta _{i}^{d}} \left | \left (\mathbf {u}_{k}^{d} \right)^{\dag } \mathbf {H}_{i}^{d} \bar {\mathbf {v}}_{i} \right |^{2}$ accounts for the interference from CUs to DR.

## Performance analysis

In the context of the quantized precoding vectors, the performance of cellular network and D2D network remains an open issue. In this section, we concentrate on the performance analysis for interference alignment with limited feedback in D2D uplink underlaying cellular networks.

As shown in last section, we consider the average rate of CU *i* as follows:

(7) $${} \begin{aligned} \mathbf{E}\left[{R_{i}^{c}}\right] =\,& \mathbf{E}\left[ \log_{2} \left(P_{c} {\eta_{i}^{c}} \left| \left(\mathbf{u}_{i}^{c}\right)^{\dag} \mathbf{H}_{i}^{c} \mathbf{v}_{i} \right|^{2} + I_{i}^{c,c} + I_{i}^{c,d} + 1 \right) \right]\\ &- \mathbf{E}\left[ \log_{2} \left(I_{i}^{c,c} + I_{i}^{c,d} +1 \right) \right]. \end{aligned}  $$

For the interference from CU *n* to CU *i*≠*n*, substituting (3) into (7), we have the following:

(8) $$ \begin{aligned} I_{i}^{c,c} &= \sum_{n \in \mathcal{Q}_{\text{CU}},n \neq i} P_{c} {\eta_{n}^{c}} \left| \left(\mathbf{u}_{i}^{c}\right)^{\dag} \mathbf{H}_{n}^{c} \mathbf{v}_{n} \right|^{2}\\ &= \sum_{n \in \mathcal{Q}_{\text{CU}}, n \neq i} P_{c} {\eta_{n}^{c}} \sin^{2}(\theta_{n}) \| \mathbf{e}_{i,n}\|^{2} \left| \bar{\mathbf{e}}_{i,n} \hat{\mathbf{v}}_{n} \right|^{2}, \end{aligned}  $$

where $\mathbf {e}_{i,n} = \left (\mathbf {u}_{i}^{c} \right)^{\dag } \mathbf {H}_{n}^{c}$ and $\bar {\mathbf {e}}_{i,n} = \mathbf {e}_{i,n}/\|\mathbf {e}_{i,n}\|$, the random variables sin2(*θ*_*n*_), ∥**e**_*i,n*_∥^2^, and $|\bar {\mathbf {e}}_{i,n}\hat {\mathbf {v}}^{2}|$ are all independent [21].

For ∥**e**_*i,n*_∥^2^, we have the following:

(9) $$ \|\mathbf{e}_{i,n}\|^{2} = \sum_{t=1}^{N} \left\| \mathbf{H}_{n}^{c} (:,t) \right\|^{2} \left\| \left(\mathbf{u}^{c}_{i}\right)^{\dag} \bar{\mathbf{H}}_{n}^{c}(:,t) \right\|^{2},  $$

where $\bar {\mathbf {H}}_{n}^{c}(:,t) = \mathbf {H}_{n}^{c}(:,t)/\left \|\mathbf {H}_{n}^{c}(:,t)\right \|$. Note that $\left \|\mathbf {H}_{n}^{c}(:,t)\right \|^{2}$ is *χ*_2_(2*M*_*c*_) distributed with variance equal to 1/2 [22]. Moreover, since $\mathbf {u}_{i}^{c}$ is of unit norm and independent of $\bar {\mathbf {H}}_{n}^{c}(:,t)$, then $\left \|\left (\mathbf {u}_{i}^{c}\right)^{\dag } \bar {\mathbf {H}}_{n}^{c}(;,t) \right \|^{2}$ is *β*(1,*M*_*c*_−1) distributed. The product of $\left \|\mathbf {H}_{n}^{c}(:,t)\right \|^{2}$ and $\left \| \left (\mathbf {u}_{i}^{c}\right)^{\dag } \bar {\mathbf {H}}_{n}^{c}(:,t) \right \|^{2}$ is exponentially (1) distributed. Based on the fact that the sum of *N* i.i.d. exponential (1) distribution random variables is *Γ*(*N*,1) distributed, we can get that ∥**e**_*i,n*_∥^2^ is *Γ*(*N*,1) distributed, it is equal to *χ*_2_(2*N*) in distribution with variance equal to 1/2.

$\forall i,n \in \mathcal {Q}_{\text {CU}}, n \neq i$, according to the theory of quantization cell approximation [19], sin2(*θ*_*n*_)∥**e**_*i,n*_∥^2^ is $\Gamma \left (N-1, 2^{-B_{n}/(N-1)}\right)$ distributed. Moreover, since $\bar {\mathbf {e}}_{i,n}$ and $\hat {\mathbf {v}}_{n}$ are i.i.d. within the same (*N*−1) dimensional of null space of **v**_*n*_, the quantity $|\bar {\mathbf {e}}_{i,n} \hat {\mathbf {v}}_{n}|^{2}$ is *β*(1,*N*−2) distributed. For the product of a $\Gamma \left (N-1, 2^{-B_{n}/(N-1)}\right)$ distributed random variable and a *β*(1,*N*−2) distributed random variable, $\left |\left (\mathbf {u}_{i}^{c}\right)^{\dag } \mathbf {H}_{n}^{c} \mathbf {v}_{n}\right |^{2}$ is exponential $\left (2^{B_{n}/(n-1)}\right)$ distributed. Thus, $P_{c}{\eta _{n}^{c}}\left |\left (\mathbf {u}_{n}^{c}\right)^{\dag } \mathbf {H}_{n}^{c} \bar {\mathbf {v}}_{n}\right |^{2}$ is exponential $\left (2^{B_{n}/(N-1)}\right)/\left (P_{c}{\eta _{n}^{c}}\right)$ distributed. Similarly, $\forall i \in \mathcal {Q}_{\text {CU}}, k \in \mathcal {Q}_{\text {DU}}$, we conclude that $P_{d}{\eta _{k}^{c}}\left |\left (\mathbf {u}_{i}^{c}\right)^{\dag } \mathbf {H}_{k}^{c} \bar {\mathbf {v}}_{k}\right |^{2}$ is exponential $\left (2^{B_{k}/(N-1)}/\left (P_{d}{\eta _{k}^{c}}\right)\right)$ distributed. Additionally, $\left |\left (\mathbf {u}_{i}^{c}\right)^{\dag }\mathbf {H}_{i}^{c} \bar {\mathbf {v}}_{i}\right |^{2}$ is *χ*_2_(2) distributed because of that $\bar {\mathbf {v}}_{i}$ and $\mathbf {u}_{i}^{c}$ are independently of $\mathbf {H}_{i}^{c}$, and $P_{c}{\eta _{i}^{c}}\left |\left (\mathbf {u}_{i}^{c}\right)^{\dag }\mathbf {H}_{i}^{c} \bar {\mathbf {v}}_{i}\right |^{2}$ is exponential $\left (1/\left (P_{c}{\eta _{i}^{c}}\right)\right)$ distributed.

$\forall i \in \mathcal {Q}_{\text {CU}}, {A_{i}^{c}} = P_{c} {\eta _{i}^{c}} \left |\left (\mathbf {u}_{i}^{c}\right)^{\dag } \mathbf {H}_{i}^{c} \bar {\mathbf {v}}_{i}\right |^{2} + I_{i}^{c,c} + I_{i}^{c,d}$ is the sum of *s*+*l* independent random variables having exponential distributions with different parameters, whose probability density function (pdf) is given by [23] the following:

(10) $$ f_{{A_{i}^{c}}}(x) = \left(\prod_{t \in \mathcal{Q}} \lambda^{c}_{i,t} \right) \sum_{k \in \mathcal{Q}} \frac{\exp\left(-x\lambda^{c}_{i,k}\right)}{\prod_{j \in \mathcal{Q}, j \neq k}\left(\lambda^{c}_{i,j}-\lambda^{c}_{i,k}\right)},  $$

where $\mathcal {Q}=\mathcal {Q}_{\text {CU}} \bigcup \mathcal {Q}_{\text {DU}}$ and

(11) $$ \lambda_{i,k}^{c} = \left\{ \begin{array}{ll} \frac{1}{P_{c} {\eta_{i}^{c}}}, & k=i, \\ 2^{\frac{B_{k}}{N-1}}\frac{1}{P_{c}{\eta^{c}_{k}}}, &k \neq i, k \in \mathcal{Q}_{\text{CU}},\\ 2^{\frac{B_{k}}{N-1}}\frac{1}{P_{d}{\eta^{c}_{k}}}, & k \in \mathcal{Q}_{\text{DU}}. \end{array} \right.  $$

Thus, we have

(12) $${} \mathbf{E}\left[ \log_{2}\left({A_{i}^{c}} + 1\right)\!\right] \!= \!\frac{\prod_{t \in \mathcal{Q}}\lambda^{c}_{i,t}}{\ln2}\!\sum_{k \in \mathcal{Q}}\!\frac{-\exp\left(\lambda^{c}_{i,k}\right)e_{i}\left(-\lambda^{c}_{i,k}\right)} {\lambda^{c}_{i,k}\prod_{j \in \mathcal{Q},j \neq k}\left(\lambda^{c}_{i,j}-\lambda^{c}_{i,k}\!\right)},  $$

where $e_{i}(x)=\int _{-\infty }^{x} e^{\alpha } \alpha ^{-1}d\alpha $ is the exponential integral function.

Note that ${G_{i}^{c}} = I_{i}^{e,c} + I_{i}^{e,d}$ is similar to ${A_{i}^{c}}$ except the absence of the term $P_{c}{\eta ^{c}_{i}} \left |\left (\mathbf {u}_{i}^{c}\right)^{\dag } \mathbf {H}_{i}^{c} \bar {\mathbf {v}}_{i} \right |^{2}$. Hence, the expectation of log2(*G*+1) can be given by the following:

(13) $$ \begin{aligned}{} \mathbf{E}\left[ \log_{2}\left({G_{i}^{c}} + 1\right)\right] =& \frac{\prod_{t \in \mathcal{Q},t \neq i}\lambda^{c}_{i,t}}{\ln2}\\&\sum_{k \in \mathcal{Q},k \neq i}\frac{-\exp\left(\lambda^{c}_{i,k}\right)e_{i}\left(-\lambda^{c}_{i,k}\right)} {\lambda^{c}_{i,k}\prod_{j \in \mathcal{Q},j \neq k,i}\left(\lambda^{c}_{i,j}-\lambda^{c}_{i,k}\right)}. \end{aligned}  $$

Now, we can get the average throughput of CU *i* as follow:

(14) $$ \mathbf{E}\left[ {R_{i}^{c}} \right] = \frac{\prod_{t \in \mathcal{Q}}\lambda^{c}_{i,t}}{\ln2}\sum_{k \in \mathcal{Q}}\frac{-\exp\left(\lambda^{c}_{i,k}\right)e_{i}\left(-\lambda^{c}_{i,k}\right)} {\lambda^{c}_{i,k}\prod_{j \in \mathcal{Q},j \neq k}\left(\lambda^{c}_{i,j}-\lambda^{c}_{i,k}\right)},  $$

and the throughput of cellular network is

(15) $${} \mathbf{E}\left[ R_{\Sigma}^{c} \right] \,=\, \sum_{i \in \mathcal{Q}_{\text{CU}}} \left(\!\frac{\prod_{t \in \mathcal{Q}}\lambda^{c}_{i,t}}{\ln2}\!\sum_{k \in \mathcal{Q}}\frac{-\exp\left(\lambda^{c}_{i,k}\right)e_{i}\left(-\lambda^{c}_{i,k}\right)} {\lambda^{c}_{i,k}\prod_{j \in \mathcal{Q},j \neq k}\left(\lambda^{c}_{i,j}-\lambda^{c}_{i,k}\!\!\right)} \right).  $$

Next, we examine the performance of D2D network. As mentioned in last section, with the precoding vectors received from BS, DR can eliminate the interference from DUs perfectly. Hence, the performance of each DU is only degraded by the interference from CUs, which is treated as noise. Therefore, we obtain the average rate of DU *k* as follows:

(16) $$ \begin{aligned} \mathbf{E}\left[ {R_{k}^{d}} \right] =\,& \mathbf{E}\left[ \log_{2}\left(P_{d}{\eta_{k}^{d}} \left| \left(\mathbf{u}_{k}^{d}\right)^{\dag}\mathbf{H}_{k}^{d}\bar{\mathbf{v}}_{k} \right|^{2} + I_{k}^{d,c} + 1 \right) \right] \\&- \mathbf{E}\left[\log_{2}\left(I_{k}^{d,c}+1\right) \right], \end{aligned}  $$

where

(17) $$ I_{k}^{d,c} = \sum_{i \in \mathcal{Q}_{\text{CU}}}P_{c}{\eta_{i}^{d}} \left| \left(\mathbf{u}^{d}_{k}\right)^{\dag} \mathbf{H}_{i}^{d} \bar{\mathbf{v}}_{i} \right|^{2}  $$

is the interference from CUs to DU *k*. Especially, for the interference from CU *i* to DU *k*, we have the following:

(18) $$ \left| \left(\mathbf{u}_{k}^{d}\right)^{\dag} \mathbf{H}_{i}^{d} \bar{\mathbf{v}}_{i} \right|^{2} = \left|\mathbf{h}_{i}^{d} \mathbf{T}^{d}_{k,i}\right|^{2} =\left\|\mathbf{h}_{i}^{d}\right\|^{2} \left|\bar{\mathbf{h}}_{i}^{d} \mathbf{T}^{d}_{k,i}\right|^{2},  $$

where $\mathbf {h}_{i}^{d} = \textmd {vec}\left (\mathbf {H}_{i}^{d}\right)$, $\bar {\mathbf {h}}_{i}^{d} = \mathbf {h}_{i}^{d} / \left \|\mathbf {h}_{i}^{d}\right \|$, $\mathbf {T}_{k,i}^{d} = \mathbf {u}_{k}^{d} \otimes \bar {\mathbf {v}}^{\dag }$, and $\left \|\mathbf {T}^{d}_{k,i}\right \| = 1$. Similar with the last subsection, $\left \|\mathbf {h}_{i}^{d}\right \|^{2}$ is *χ*_2_(2*NM*_*d*_) distributed with variance equal to 1/2. Moreover, $\left |\bar {\mathbf {h}}_{i}^{d}\mathbf {T}_{k,i}^{d}\right |^{2}$ is *β*(1,*NM*_*d*_−1) distributed because that $\mathbf {T}_{k,i}^{d}$ of unit norm is independent of $\bar {\mathbf {h}}_{i}^{d}$. As the product of a *χ*_2_(2*NM*_*d*_) distributed random variable and a *β*(1,*NM*_*d*_−1) distributed random variable, $\left |\left (\mathbf {u}_{k}^{d}\right)^{\dag } \mathbf {H}_{i}^{d} \bar {\mathbf {v}}_{i}\right |$ is exponential (1) distributed, and $P_{c}{\eta _{i}^{d}} \left |\left (\mathbf {u}_{k}^{d}\right)^{\dag } \mathbf {H}_{i}^{d} \bar {\mathbf {v}}_{i}\right |$ is exponential $\left (1/\left (P_{c}{\eta _{i}^{d}}\right)\right)$ distributed. Similarly, the desired signal power $P_{d}{\eta _{k}^{d}}\left |\left (\mathbf {u}_{k}^{d}\right)^{\dag }\mathbf {H}_{k}^{d}\bar {\mathbf {v}}_{k}\right |^{2}$ is exponential $\left (1/\left (P_{d}{\eta _{k}^{d}}\right)\right)$ distributed.

$\forall k \in \mathcal {Q}_{\text {DU}}$, ${A_{k}^{d}} = P_{d}{\eta _{k}^{d}}\left |\left (\mathbf {u}_{k}^{d}\right)^{\dag } \mathbf {H}_{k}^{d} \bar {\mathbf {v}}_{d}\right |^{2} + I_{k}^{d,c}$ is the sum of *s*+*l* independent random variables having exponential distributions with different parameters, whose pdf is given by the following:

(19) $$ f_{{A_{k}^{d}}}(x) = \left(\prod_{t \in \mathcal{Q}_{\text{CU},k}} {\nu_{t}^{d}} \right) \sum_{i \in \mathcal{Q}_{\text{CU},k}}\frac{\exp\left(-x{\nu_{i}^{d}}\right)} {\prod_{j \in \mathcal{Q}_{\text{CU},k},j \neq i}\left({\nu_{j}^{d}} - {\nu_{i}^{d}}\right)},  $$

where $\mathcal {Q}_{\text {CU},k} = \mathcal {Q}_{\text {CU}} \bigcup \{ k \in \mathcal {Q}_{\text {DU}}\}$, and

(20) $$ {\nu_{i}^{d}} = \left\{ \begin{array}{ll} \frac{1}{P_{c} {\eta_{i}^{d}}}, & i \in \mathcal{Q}_{\text{CU}}, \\ \frac{1}{P_{d} {\eta_{i}^{d}}}, & i \in \mathcal{Q}_{\text{DU}}. \end{array} \right.  $$

Therefore, we have the following:

(21) $$ \begin{aligned} \mathbf{E}\left[ \log_{2} \left({A_{k}^{d}} + 1\right) \right] =& \frac{\prod_{t \in \mathcal{Q}_{\text{CU},k}}{\nu_{t}^{d}}}{\ln2}\\ &\sum_{i \in \mathcal{Q}_{\text{CU},k}} \frac{-\exp\left({\nu_{i}^{d}}\right)e_{i}\left(-{\nu_{i}^{d}}\right)}{\prod_{j \in \mathcal{Q}_{\text{CU},k},j \neq i} \left({\nu_{j}^{d}} - {\nu_{i}^{d}}\right)}. \end{aligned}  $$

Note that ${G_{k}^{d}} = I_{k}^{d,c}$ is similar to ${A_{k}^{d}}$ except the absence of the term $P_{d}{\eta _{k}^{d}}\left |\left (\mathbf {u}_{k}^{d}\right)^{\dag } \mathbf {H}_{k}^{d} \bar {\mathbf {v}}_{k}\right |^{2}$, so the expectation of $\log _{2}\left ({G_{k}^{d}} +1\right)$ can be given by the following:

(22) $$ \begin{aligned} \mathbf{E}\left[ \log_{2} \left({G_{k}^{d}} + 1\right) \right] =& \frac{\prod_{t \in \mathcal{Q}_{\text{CU}}}{\nu_{t}^{d}}}{\ln2}\\ &\sum_{i \in \mathcal{Q}_{\text{CU}}} \frac{-\exp\left({\nu_{i}^{d}}\right)e_{i}\left(-{\nu_{i}^{d}}\right)}{{\nu_{i}^{d}} \prod_{j \in \mathcal{Q}_{\text{CU}},j \neq i} \left({\nu_{j}^{d}} - {\nu_{i}^{d}}\right)}. \end{aligned}  $$

Now, we can obtain the average throughput of DU *k* as follows:

(23) $${} \mathbf{E}\left[ {R_{k}^{d}} \right] = \frac{\prod_{t \in \mathcal{Q}_{\text{CU},k}}{\nu_{t}^{d}}}{\ln2} \sum_{i \in \mathcal{Q}_{\text{CU},k}} \frac{-\exp\left({\nu_{i}^{d}}\right)e_{i}\left(-{\nu_{i}^{d}}\right)}{{\nu_{k}^{d}} \prod_{j \in \mathcal{Q}_{\text{CU}},j \neq i} \left({\nu_{j}^{d}} - {\nu_{i}^{d}}\right)},  $$

and the throughput of D2D network is as follows:

(24) $${} {\fontsize{8.6pt}{9.6pt}\begin{aligned} \mathbf{E}\left[ R_{\Sigma}^{d} \right] =\! \sum_{k \in \mathcal{Q}_{\text{DU}}} \!\!\left(\frac{\prod_{t \in \mathcal{Q}_{\text{CU},k}}{\nu_{t}^{d}}}{\ln2} \!\sum_{i \in \mathcal{Q}_{\text{CU},k}} \frac{-\exp\left({\nu_{i}^{d}}\right)e_{i}\left(-{\nu_{i}^{d}}\right)}{{\nu_{k}^{d}} \prod_{j \in \mathcal{Q}_{\text{CU}},j \neq i} \left({\nu_{j}^{d}} - {\nu_{i}^{d}}\right)}\!\right). \end{aligned}}  $$

For interference alignment with limited feedback, the performance of system degraded significantily due to the residual interference caused by the imperfect CSI. To optimize for the design, analysis, and deployment of the systems, the analytic expression of the average throughput of the D2D communication networks is a key factor and is very important. Howerver, as far as I konw, the performance analysis is still an open issue, so it is a basic work for the study of D2D communications.

## Numerical results

In this section, we evaluate the analytic results of interference alignment with limited feedback in a D2D uplink underlay cellular network. Without losing generality, we set *N*=3, *M*_*c*_=5, *M*_*d*_=4, *s*=3, and *l*=4. We focus on a single cellular cell which contains an BS near its center, and a DR which is 0.5*r* m away from the BS, where *r* is the radius of the cellular cell. The *s* CUs are distributed randomly in the cellular cell. The distance between *l* DUs and DR distribute randomly between 0.05*r* and 0.45*r*, and we configure *r* to be 300 m. The channel is assumed to obey a frequency flat block-fading channel model. We take the average of sum rate as the performance metric and evaluate the precision of our analytic results by means of Monte Carlo simulations (averaging over 1000 realizations) with random generated small-scale fading, which is generated so that the entries of the matrices are i.i.d. according to $\mathcal {CN}(0,1)$. Moreover, we use the path loss model recommended by ITU-R as shown by the following:

(25) $$ \eta = 40 \log_{10} {\alpha} + 30 \log_{10} f + 49 [\text{dB}],  $$

where *α* is the distance in km and *f* is the operating frequency in MHz which is set to be 2 GHz. Moreover, the noise power is set to be −113 dBm.

**Fig. 2:**
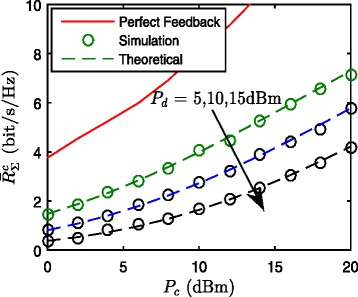
Theoretical and simulation $\bar {R}_{\Sigma }^{c} = \mathbf {E}\left [R_{\Sigma }^{c}\right ]$ with different transmit powers of DUs

**Fig. 3:**
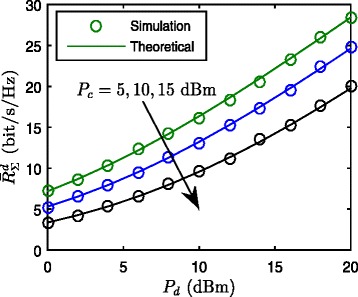
Theoretical and simulation $\bar {R}_{\Sigma }^{d} =\mathbf {E}\left [R_{\Sigma }^{d}\right ]$ with different transmit powers of DUs

In Fig. 2, we plot both the empirical sum of average rate of cellular network (from simulation) and the analytical results in (15), for the transmit power of DUs *P*_*d*_=5,10,15 dBm with the number of feedback bits *B*_*i*_=10 for each DUs and CUs. The derived analytical sum of average rate of D2D network in (24) and the simulation results are also plotted in Fig. 3. The numerical results show that the derived close-form expressions are sufficiently accurate in the sum of the average rate of CUs and DUs achieved by interference alignment with limited feedback in D2D uplink underlaying cellular networks. It is noted that, by comparing the Figs. 2 and 3, we can find that the sum rate of DUs is much larger than that of CUs. The performance gap is primarily attributed to the fact that DUs stand closer to DR than CUs on average, which leads to stronger desired signals and weaker interferences.

## Conclusions

In this work, we comprehensively investigate the interference alignment scheme with limited feedback for D2D underlaying cellular networks. We derive the average sum rate of cellular network and D2D network analytically. Thus, the exact performance can be obtained for a given path loss, transmit SNR, and feedback bits. The analysis results provide some basis for the design and optimization for D2D communication networks.
